# Positive in vitro wound healing effects of functional inclusion bodies of a lipoxygenase from the Mexican axolotl

**DOI:** 10.1186/s12934-018-0904-0

**Published:** 2018-04-07

**Authors:** Anne Stamm, Sarah Strauß, Peter Vogt, Thomas Scheper, Iliyana Pepelanova

**Affiliations:** 1 Institute of Technical Chemistry, Gottfried Wilhelm Leibniz University, Callinstraße 5, 30167 Hannover, Germany; 2 Department of Plastic, Aesthetic, Hand and Reconstructive Surgery, Hannover Medical School, Carl-Neuberg-Straße 1, 30625 Hannover, Germany

**Keywords:** AmbLOXe, Axolotl (*Ambystoma mexicanum)*, Active inclusion bodies, In vitro wound healing assay, Lipoxygenase, Nanopills

## Abstract

**Background:**

AmbLOXe is a lipoxygenase, which is up-regulated during limb-redevelopment in the Mexican axolotl, *Ambystoma mexicanum*, an animal with remarkable regeneration capacity. Previous studies have shown that mammalian cells transformed with the gene of this epidermal lipoxygenase display faster migration and wound closure rate during in vitro wound healing experiments.

**Results:**

In this study, the gene of AmbLOXe was codon-optimized for expression in *Escherichia coli* and was produced in the insoluble fraction as protein aggregates. These inclusion bodies or nanopills were shown to be reservoirs containing functional protein during in vitro wound healing assays. For this purpose, functional inclusion bodies were used to coat cell culture surfaces prior cell seeding or were added directly to the medium after cells reached confluence. In both scenarios, AmbLOXe inclusion bodies led to faster migration rate and wound closure, in comparison to controls containing either no AmbLOXe or GFP inclusion bodies.

**Conclusions:**

Our results demonstrate that AmbLOXe inclusion bodies are functional and may serve as stable reservoirs of this enzyme. Nevertheless, further studies with soluble enzyme are also necessary in order to start elucidating the exact molecular substrates of AmbLOXe and the biochemical pathways involved in the wound healing effect.

## Background

The number of patients suffering from chronic wounds is steadily increasing due to demographic changes and the upward trend of diabetic cases. Wound healing has thus become one of the major challenges of modern medicine with rising patient numbers and costs. According to estimates, 2.2 million patients in Great Britain suffer from chronic wounds, leading to estimated costs of 5.3 billion £ [1]. Estimations for Germany amount to comparable numbers with 2–4 million patients with chronic wounds, connected to healthcare costs of around 4 billion € [2]. Numerous medical products have been developed to support and improve wound healing with respect to the control of wound moisture, antibiosis and the application of growth factors. Despite all these innovations, wound healing is still frequently associated with complications, a slow healing process and scarring.

In the context of regeneration, scientists have been fascinated by the axolotl (*Ambystoma mexicanum*) and its regenerative capacity since 1863, when French scientists brought a group of 34 animals to Paris from a Mexico expedition. The animals mated in captivity and founded the first population in Europe [3]. Although regeneration can also fail in axolotls, their healing capacity is significant and functional even in old animals, which stands in strong contrast to other vertebrates, especially adult mammals. In 2011 Menger et al. described the up-regulation of a gene named AmbLOXe, observed during limb regeneration of the axolotl [4]. Database analysis suggests a relationship to the family of lipoxygenases. This is a group of dioxygenases which catalyze the oxygenation of polyunsaturated fatty acids [5]. In humans, at least six genes (ALOX5, ALOX 12, ALOX12B, ALOX15, ALOX 15B, ALOXE3) coding for lipoxygenases (5-LOX, pll12-LOX, 12R-LOX, 12/15-LOX, 15-LOX2, eLOX3) have been described. The lipoxygenase metabolism is quite diverse and complex. Due to this diversity, metabolites of lipoxygenase-catalyzed reactions are involved in numerous processes in various tissue and cell types e.g. wound healing, mediation of inflammation, modulation of platelet aggregation, tumor suppression, tumor progression and adipocyte differentiation [5, 6].

Menger et al. were able to clone the AmbLOXe gene from the Mexican salamander and experiment with its effect on transfected mammalian cell lines. Human cell lines showed a significantly accelerated wound closure in in vitro scratch assays after transfection with AmbLOXe [4]. An in vivo study was also performed, by treating full-thickness wounds in the back of mice with mouse fibroblasts transfected with AmbLOXe. In these animal studies, accelerated wound healing was also observed, in contrast to treatment with cells carrying only an empty vector or a vector coding for human 12R lipoxygenase [7]. However, these results should be approached with caution, as literature queries the suitability of murine models for predicting similar effects in humans, as both species exhibit profound differences in lipoxygenase metabolism regarding substrates and products [6]. Furthermore, the molecular cascade, substrates and metabolites of AmbLOXe have not yet been described. Menger et al. postulated an affiliation to epidermal lipoxygenases based on sequence clustering with human epidermal lipoxygenases [4], but a profound proof by metabolite and X-ray analysis of crystals is still pending. For additional characterization of AmbLOXe, high amounts of isolated enzyme are required, which cannot be directly extracted from the Mexican salamander alone. Therefore, an efficient expression system is necessary, which can deliver functional AmbLOXe.

For this purpose, we cloned the AmbLOXe gene in *E. coli* and overexpressed the protein in the form of inclusion bodies. One common strategy to obtain bioactive enzyme from this stage is the solubilization of the insoluble protein and the screening of suitable refolding conditions [8]. However, before attempting solubilization and refolding, we hypothesized that these inclusion bodies may display some functionality as nanopills have been shown to do so [9, 10]. It has been reported in literature, that overexpressed recombinant protein may exist in an amorphous, porous structure which may function as a natural immobilized biocatalyst [11]. It has been shown by several research groups, for enzymes, growth factors, as well as for fluorescent proteins, that inclusion bodies exhibit functionality [12–14].

The exact mechanism of action of functional inclusion bodies has not been precisely elucidated. Free release of the correctly-folded and active protein from the nanopill reservoir surely plays a role, but also bacterial IBs are readily internalized by mammalian cells [15]. Research suggests that cellular intake works predominantly by cell-membrane association of amyloid particles followed by macropinocytosis [16]. A large proportion of the engulfed amyloid deposits are degraded, but a significant part of bioactive protein is released into the cytosol, probably assisted by the action of contaminant microbial chaperones and endogenous mammalian chaperones [16, 17].

There are numerous advantages to use inclusion bodies; they can be easily isolated and purified in large amounts. In addition, they are mechanically stable and can be easily immobilized on surfaces. In the case of AmbLOXe, it is of great interest to provide a form acting as a stable reservoir of the enzyme, which can then be tailored for therapeutic applications in dressings for chronic wounds.

Previously, it has been shown that mammalian cells transfected with AmbLOXe exhibit faster wound closure rate. In this study, we tested the effect of the recombinant enzyme on in vitro cell culture directly, by adapting a wound healing assay to test the hypothesis that AmbLOXe inclusion bodies can act as a functional reservoir of the enzyme and can influence wound healing in vitro. In order to be able to place AmbLOXe effects in perspective, we also tested GST-GFP inclusion bodies as a negative control, and the epidermal lipoxygenase ALOXe3 from *Homo sapiens*, which displays the highest sequence homology to the AmbLOXe enzyme in humans.

## Methods

All chemicals used in this study were purchased either from Sigma Aldrich (St. Louis, USA) or Carl Roth (Karlsruhe, Germany), unless indicated otherwise. Aqueous solutions were prepared using deionized water from an Arium^®^ water purification system (Sartorius-Stedim Biotech, Göttingen, Germany). pH values were adjusted at 25 °C using a HI 221 pH meter (Hanna Instruments, Québec, Canada). Optical density was determined at 600 nm using a Multiskan™ GO spectrophotometer (Thermo Fisher, Waltham, USA).

### Cloning and expression

The cDNA GenBank sequences for ALOXe3 (NM_021628) and AmbLOXe (EU814616) were carefully codon-optimized for expression in *Escherichia coli* K12 derivative strains, taking into account the GC content and codon usage in the host organism compared to the organism of origin. These codon-optimized sequences were completed by adding a NcoI restriction site, a His-Tag and a TEV cleavage site, all separated by small spacers at the N-terminus as well as a XhoI restriction site at the C-terminus. The constructs for ALOXe3 (2223 bp) and AmbLOXe (1959 bp) were produced by GeneArt™ gene synthesis service (Thermo Fisher, Waltham, USA). The synthetic genes were delivered in a pMK-RQ vector construct. Utilizing the added restrictions sites NcoI and XhoI, the constructs were cloned individually in frame into the cloning/expression region of a pET-28b(+) (Merck Millipore, Billerica, USA) vector. The correct cloning was verified by sequencing the cloning/expression region of both vectors (pET-28b_ALOXe3 and pET28b_AmbLOXe) using the T7 promotor primer and the T7 terminator primer, as well as primers covering the middle part of both sequences (mid_ALOXe3: GGCATCTCTGGGCATGAAACTG and mid-AmbLOXe: TGCAGAAGGGTAACATCTACATC) (Eurofins Genomics, Ebersberg, Germany). Positive constructs were transformed into *E. coli* BL21(DE3) cells (New England Biolabs, Ipswich, USA).

Inclusion bodies of GFP with a GST (glutathione-S-transferase) tag were used as a negative control in this study. GFP is a highly soluble protein on its own, but the GST-tag increases its propensity to form IBs during heterologous expression. GST-GFP inclusion bodies were produced in a *E. coli* BL21 (DE3) strain containing the pETM30-His6-GST-GFP vector as described elsewhere [18].

Cultivation for inclusion body production was performed in LB (lysogeny broth)-medium (10 g l^−1^ tryptone, 5 g l^−1^ yeast extract, 10 g l^−1^ sodium chloride) containing kanamycin (30 μg ml^−1^) as an antibiotic for selection. Pre-cultures were set up in 25 ml LB-medium which was inoculated with glycerol stocks of the corresponding cells and incubated overnight at 30 °C and 180 rpm. Main cultures in a 2-l flask with a cultivation volume of 500 ml were inoculated with pre-culture to OD_600_ = 0.05 and bacteria were grown at 37 °C and 200 rpm using a KS 4000 I control shaker (IKA, Staufen, Germany). At OD_600_ = 0.7, the temperature was lowered to 25 °C and protein production was induced by adding 0.5 mM Isopropyl β-d-1-thiogalactopyranoside (IPTG; VWR, Darmstadt, Germany). Samples were taken before induction and 3 h after induction when the cultivation was stopped.

### Bacterial cell disruption/inclusion body purification

After the cultivation was stopped, 2 μM phenylmethane sulfonyl fluoride (PMSF), 100 μg ml^−1^ lysozyme and one cOmplete™ EDTA-free protease inhibitor cocktail tablet (Sigma-Aldrich, St. Louis, USA) were added to the culture broths and shaken (2 h, 37 °C, 180 rpm). The culture broths were then filled into sterile 1 l Nalgene^®^ PPCO square bottles (Thermo Fisher, Waltham, USA) and frozen overnight at − 80 °C. After defrosting in a water bath at 22 °C, 2 ml sterile Triton X-100 (Serva Electrophoresis GmbH, Heidelberg, Germany) was added and the solutions were incubated (1 h, room temperature, 50 rpm). 100 μl of this solution was spread on a LB-agar plate with no antibiotics to check for any viable bacterial cells. (Note: at this point there were already no bacterial cells viable for all three (ALOXe3, AmbLOXe and GST-GFP) inclusion body purifications). After another freeze (− 80 °C) and thaw (22 °C) cycle, 125 μl sterile Nonidet P40 was added and incubated (1 h, 4 °C, 50 rpm). 1 μg ml^−1^ DNase (AppliChem, Darmstadt, Germany) as well as 1 mM MgSO_4_ were added to the solutions and shaken (1 h, 37 °C, 180 rpm). The culture broth was then centrifuged (15 min, 4 °C, 15,000×*g*). The resulting pellet was washed with 25 ml sterile buffer (50 mM Tris–HCl (pH 8), 100 mM NaCl, 1 mM EDTA, 0.5% Triton X-100) and centrifuged again under the same conditions. The supernatant was discarded and the pellet containing the inclusion bodies was resuspended in 25 ml phosphate buffered saline (PBS) and again tested for sterility on a LB-agar plate with no antibiotics and frozen overnight at − 80 °C. The suspension was again defrosted in a 22 °C water bath, centrifuged (15 min, 4 °C, 15,000×*g*), the supernatant was discarded and purified inclusion bodies were resuspended in 25 ml ultrapure water. The inclusion body solutions were aliquoted, centrifuged (15 min, 4 °C, 15,000×*g*), the supernatant was discarded and aliquots were stored at − 80 °C until further use. Before using these aliquots in cell culture, they were again tested for sterility on LB-agar plates, as well as for sterility in cell culture media containing no antibiotics. Additionally, the concentration of enzyme was determined using densitometry and samples were tested for confirmation of protein identity by using LC–MS (data not shown).

### Determination of inclusion body concentration

Samples of purified protein, as well as a BSA standard ranging from 0.04 to 0.5 mg ml^−1^ (Pierce™ bovine serum albumin standard ampules, 2 mg ml^−1^; Thermo Fisher, Waltham, USA) and an unstained protein molecular weight marker (#26610; Thermo Fisher, Waltham, USA) were applied in SDS-PAGE analysis (10% Mini-PROTEAN^®^ TGX Stain-Free™ protein gels, 15 wells, #4568036; Bio-Rad Laboratories, Hercules, USA). Gels were stained by colloidal Coomassie staining. The quantity of proteins in the final aliquots was determined by densitometry using ImageJ software (NIH, USA).

### Characterization of inclusion bodies

The size distribution and zeta potential of the IBs used in this study were analyzed with a Litesizer™ 500 device (Anton Paar, Graz, Austria). Samples were resuspened in ultrapure water and were treated with a Labsonic^®^M sonicator for 1 min, with a 0.5 pulse regime at 40% amplitude. Fresh suspensions were always prepared prior analysis and transferred to single use cuvettes for dynamic light scattering (DLS) measurements, or alternatively, to omega cuvettes (Anton Paar, Graz, Austria) for the zeta potential measurements. Samples were analyzed in triplicates, averaging 30 single measurements.

### Cell culture

HaCaT keratinocytes (300493; CLS Cell Lines Services, Eppelheim, Germany) were cryoconserved, revitalized and cultivated as recommended by the distributor. Cells were cultured in a Dulbecco’s Modified Eagle’s Medium (DMEM; Sigma-Aldrich, St. Louis, USA) supplemented with 4.5 g l^−1^ glucose, 2 mM l-glutamine and 10% fetal bovine serum (FBS; Biochrom, Berlin, Germany) at 37 °C and 5% CO_2_, culture medium was renewed every 2–3 days and cells were passaged once a week (split ratio 1:6). Before detaching the cells with trypsin/EDTA (Sigma-Aldrich, St. Louis, USA) for subculturing, they were incubated with 0.05% EDTA solution for 10 min at 37 °C to promote detachment. Additionally, cells were vortexed after detachment and filtered through a Faclon™ Cell Strainer (40 μm; Corning, Corning, USA) after resuspension, in order to obtain a single-cell suspension for further seeding.

### Wound healing assay using cell culture inserts

Wound healing assays were performed by decoration of the cell culture surface (IB coating) or by direct addition of inclusion bodies to the culture medium. In wound healing experiments with IB coating, inclusion bodies were added to the cell culture surface before cells were seeded. Briefly, inclusion bodies aliquots were defrosted, resuspended in 0.15 M sodium chloride solution and further diluted, leading to a final concentration of 0.2 μg ml^−1^. 70 μl of this solution was added to each of the two compartments of a silicone insert (culture-insert, 2-wells 24-well plate, ibiTreat; Ibidi, Martinsried, Germany), resulting in a final IB coating density of 62.5 ng·cm^−2^. The coating was incubated for 2–3 h at 4 °C. The supernatant was removed and cells were seeded with a cell density of 2.4 × 10^5^ cells cm^−2^. Cells were grown and after 24 h the medium was changed to starvation medium containing only 1% FBS. Cells were grown further and after 18 h the culture-inserts were removed using sterile tweezers resulting in a 500 μm-wide gap. More starvation medium was added to fill the whole well of the 24-well plate.

For experiments with IB addition to the cell culture media, cells were directly seeded to the cell culture surface and grown as described above. After culture-inserts’ removal, the starvation medium was changed with starvation medium containing inclusion bodies resulting in the same final concentration (62.5 ng cm^−2^) as described above.

### Analysis

The subsequent healing process was recorded using a LumaScope 600 microscope (Etaluma, Carlsbad, USA) for one well exemplarily. Additionally, images of the starting conditions (~ 500 μm gaps) for at least 2–3 wells were taken and all wells of the 24-well plate were checked visually for any irregularities. 12 h after wounding, cell growth was stopped and cells were fixed using paraformaldehyde. Fixed cells were stained using trypan blue and micrographs of all wells for wound size measurements were taken (Olympus IX50 inverted microscope with cellSens Standard software; Olympus, Hamburg, Germany). Wound sizes were determined using ImageJ software with the MRI wound healing tool macro (http://dev.mri.cnrs.fr/projects/imagej-macros/wiki/Wound_Healing_Tool). Images were converted into 32-bit grayscale and default settings (method: variance, variance filter radius: 10, threshold: 50, radius open: 4, min size: 10,000) were used. Wound sizes were determined for three replicates for each protein (ALOXe3, AmbLOXe and GST-GFP) and for each condition (coating and direct addition to culture media). The mean value and standard deviation were calculated.

### CellTiter-Blue^®^ (CTB) analysis of cell viability

For toxicity testing of IBs and the detection of possible effects on cell proliferation, a CTB assay (CellTiter-Blue^®^ Cell Viability Assay, Promega, Madison, USA) was performed according to the manufacturer’s protocol. Briefly, HaCaT cells were seeded at a cell density of 3 × 10^4^ cells cm^−2^ in a 96-well plate and grown for 24 h as described above. The medium was changed to starvation medium containing only 1% FBS and cells were grown for further 18 h. Inclusion bodies (GST-GFP, ALOXe3 and AmbLOXe) were either added immobilized on cell culture surfaces before cell seeding or were added directly to the medium after the 18 h starvation phase. The concentration of IBs was 62.5 ng cm^−2^ in both cases. The end of the 18 h starvation phase was set as the starting point and CTB analysis was performed 24 and 72 h after this time point for both the coating setup, as well as the addition of IBs setup. For the CTB assay, the media was removed and 100 μl of a 10% CTB solution in DMEM were added to each well and incubated for 1 h at 37 °C. The fluorescence signals at an excitation wavelength of 544 nm and an emission wavelength of 590 nm were determined using a microplate reader (Fluoroskan Ascent, Thermo Fisher Scientific, Waltham, USA). Each sample was measured in triplicates. Wells containing only cells and no IBs were included as a control.

## Results and discussion

### Expression and purification of functional inclusion bodies from bacterial cells

The DNA constructs using a pET28b(+) plasmid as a backbone were created successfully using standard tools of genetic engineering. Constructs were first transformed into *E. coli* TOP10 cells for plasmid propagation. DNA plasmids were then checked for sequence accuracy using a sequencing service. Sequence-confirmed plasmids were finally transformed into *E. coli* BL21(DE3) cells for production of heterologous protein.

**Fig. 1:**
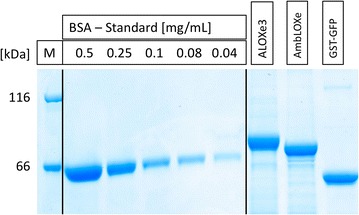
Concentration determination using densitometry; ALOXe3 (83 kDa), AmbLOXe (74 kDa), GST-GFP (54 kDa)

Inclusion bodies could be successfully produced and purified from *E. coli* culture. The concentration of ALOXe3, AmbLOXe and GST-GFP inclusion bodies was determined using densitometry. The yield of the inclusion bodies after purification based on 1 l culture broth was 175 mg for ALOXe3, 171 mg for AmbLOXe and 159 mg for GST-GFP (Fig. 1). Impurities due to other proteins which co-purified with the IBs were very low and below the detection limit of densitometry. Prior to mammalian cell culture experiments, all inclusion bodies were tested for sterility on agar plates, as well as in cell culture media.

### Size determination and zeta potential of the inclusion bodies

**Fig. 2:**
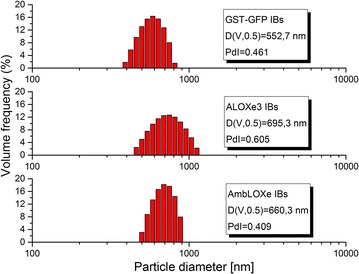
IB-size distribution for GST-GFP, ALOXe3 and AmbLOXe. The D(V,0.5) value represents the volume median particle diameter. The polydispersity (PdI) is represented as the undersize span, indicating the broadness of the size distribution

The produced inclusion bodies were analyzed with dynamic light scattering (DLS) to characterize their size and zeta potential. Figure 2 shows histograms of the size distribution of the IBs used in this study. The sizes ranged between 500 and 700 nm and are typical for inclusion bodies [19]. The lipoxygenase IBs are slightly larger than the GST-GFP IBs. The polydispersity is represented by the undersize span (calculated by (D(V,0.9) − D(V,0.1))/D(V,0.5)), which is an indicator of the broadness of the size distribution. In this case, the ALOXe3 inclusion bodies show the widest size distribution. Zeta potential measurements returned values of − 12.6 mV ± 0.4 mV (GST–GFP), 14.9 mV ± 0.3 mV (ALOXe3) and 25.7 mV ± 0.4 mV (AmbLOXe). The low zeta potential of the produced IBs |30 mV| is typical for protein inclusion bodies and reflects their tendency to agglomerate [20].

### General consideration for wound healing assays

**Fig. 3:**
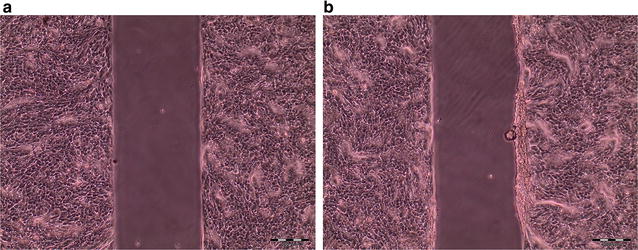
Comparison of wound area resulting after variable growth times in cultivation medium with 10% FBS, cell seeding density 2.4 × 10^5^ cells cm^−2^,  **a** cell growth for 24 h, **b** cell growth for 36 h

Repeatability is a major concern during the establishment of a wound healing assay and can be achieved by the successful application of reproducible wounding. Key factors for creating reproducible wounds are the seeding density of cells, as well as the growth time [21]. This fact is also valid for the wound healing methods with culture-inserts used in this work. In this case, a high cell seeding density (> 2.4 × 10^5^ cells cm^−2^) and/or long growth times (> 24 h) led to uneven wound edges (Fig. 3). Once suitable cell density parameters and growth times were implemented, repeatability of experiments improved considerably.

**Fig. 4:**
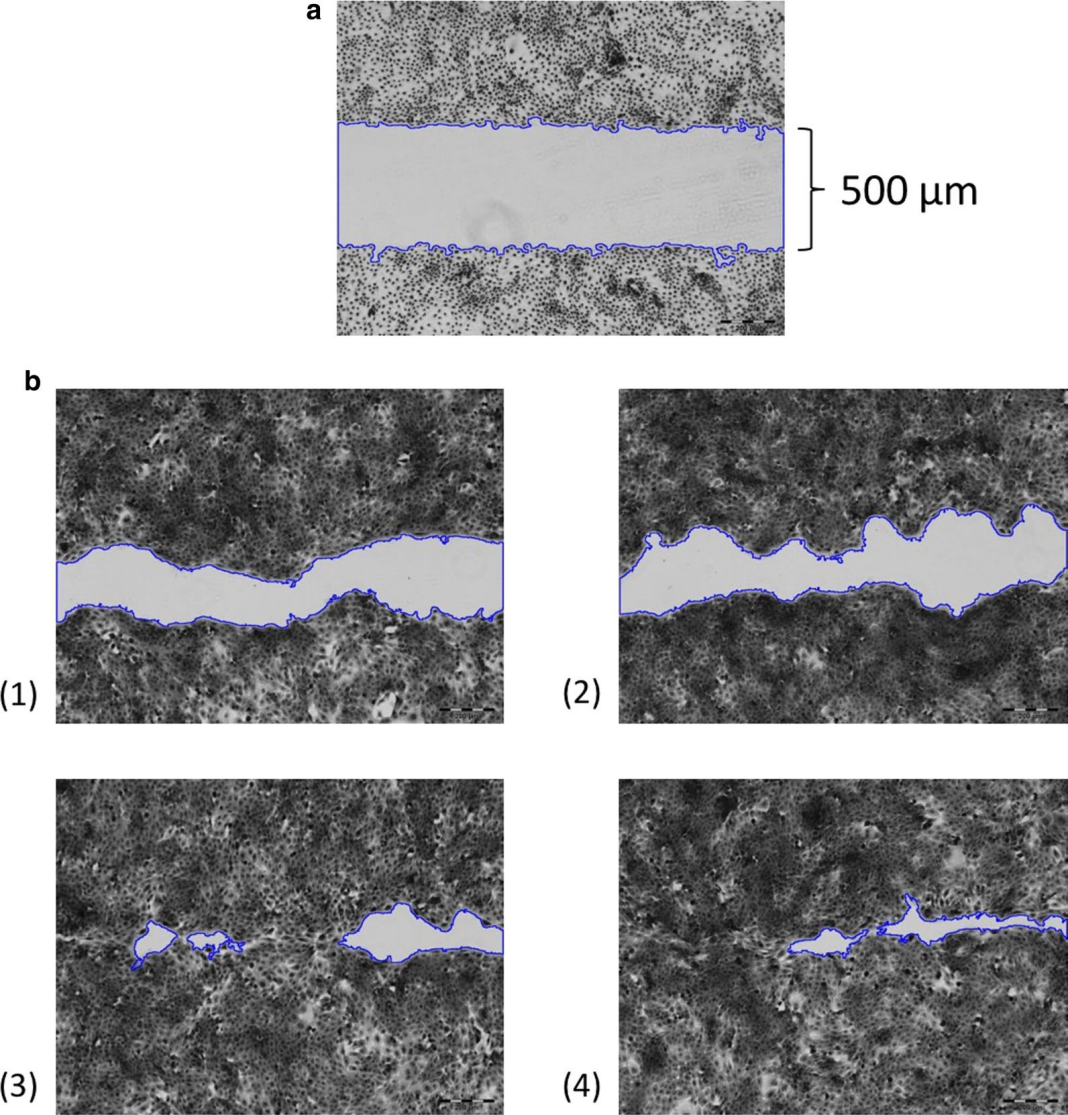
Measurement of wound areas using the MRI wound healing tool macro for ImageJ; coating with inclusion bodies (62.5 ng/cm^2^); **a** After culture-insert removal (t = 0 h); **b** After cell fixation and staining (t = 12 h), (1) control (no inclusion bodies), (2) coating with GST-GFP inclusion bodies, (3) coating with ALOXe3 inclusion bodies, (4) coating with AmbLOXe inclusion bodies

The MRI wound healing tool macro was used for wound size determination, as shown for an exemplary dataset in Fig. 4. Wound edges were detected accurately on micrograph pictures of stained cells. For better edge detection, images were converted into grayscale.

### Epidermal lipoxygenase inclusion bodies promote wound healing

**Fig. 5:**
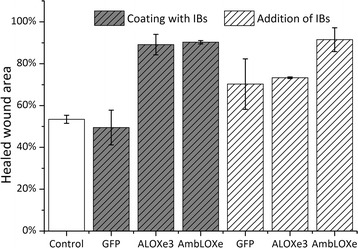
Wound closure area  % determined in the wound healing assays after 12 h; coating with inclusion bodies or direct addition of inclusion bodies to culture media (62.5 ng cm^−2^); control = no inclusion bodies

Since no signal pathways and exact substrates of AmbLOXe are currently known, a straightforward way to test the hypothesis that the inclusion bodies are functional was to test their effects in a wound healing assay. Here, two different modes of particle addition were investigated: the coating of the cell culture surfaces with inclusion bodies prior to cell seeding or the direct addition of the IBs immediately after cell wounding. IBs are used to pattern cell culture surfaces and the mechanical cues they provide support the adhesion and proliferation of mammalian cells [22, 23]. If the IBs are derived from a relevant biological protein like a cytokine, they can exert a dual role, behaving both like a bioactive agent and a topographic stimulant [24]. By including two different modes of particle addition, our experimental setup aims to investigate whether wound closure effects are based on cell stimulation by the topography of the cell culture surface, or by an inherent activity of the LOX enzymes. The results of the experiments are presented in Fig. 5. Both lipoxygenases displayed a positive effect on wound healing, with AmbLOXe showing higher efficacy in both modes of application.

In the coating experiments, 90.3% of the wound area was closed with AmbLOXe and 89.1% with ALOXe3 after 12 h. In comparison, the control wounds without coating and the negative control with GFP showed 53.4 and 49.5% wound closure, respectively. The use of GST-GFP inclusion bodies for coating showed no topographical stimulation on proliferation and cell migration at the concentration and IB size used in this study. Similar results were obtained in the setup with direct addition of inclusion bodies to the cell culture media. Addition of AmbLOXe to the cell media resulted in 91.5% wound closure after 12 h, compared to 73.3% with ALOXe3 and 70.3% with GFP. Direct addition of IBs to the media led to faster cell migration in comparison to the control for all proteins tested.

It remains unclear whether the observed effects of the AmbLOXe IBs result from IB internalization or the release of low levels of correctly-folded lipoxygenase into the medium. Incubating the inclusion bodies in cell culture medium alone for 24 h at 37 °C did not lead to the solubilization and detection of soluble protein from all three proteins tested (data not shown).

**Fig. 6:**
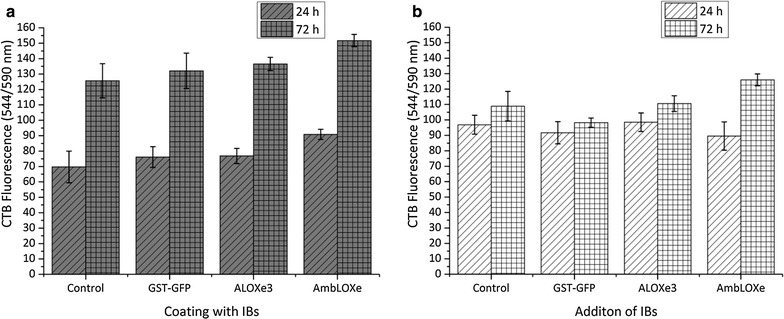
CTB assay of cell proliferation performed with different modes of IB addition: **a** coating with IBs, **b** direct addition of IBs, the control sample contained no inclusion bodies

The readout of a wound healing assay is usually carried out after 12 h, in order to differentiate migration from overall cell proliferation effects [25]. An interesting side effect of the experimental design and the two various modes of IB addition is that the cells spend different times in contact with the IBs. In the coating experiment, cells spend a total of 54 h in contact with the inclusion bodies (24 h growth phase, 18 h starvation phase, 12 h wound healing phase). In the direct addition setup, cells are exposed to the IBs for 12 h only, which is the duration of the wound healing phase. It is interesting to speculate whether the longer contact times in the coating experiment lead to a stimulation of cell proliferation, which then indirectly caused higher migration readout during the wound healing phase. In order to elucidate the effects of IBs on the cells, we performed a CTB cell viability assay with both modes of IB exposure (direct addition and coating). As can be seen from Fig. 6, the lack of toxicity for all IBs could be demonstrated for periods longer than the exposure times of the wound healing assay. The CTB assay also revealed that there are no significant differences in cell proliferation between the control (no IB coating) and the IBs after 24 and 72 h (Fig. 6a). This outcome confirms that the results observed in the wound healing assay are based mainly on migration effects caused by exposure to AmbLOXe and are not caused solely by increased cell proliferation.

## Conclusion

In this work we could adapt an in vitro wound healing assay to investigate the effect of epidermal lipoxygenase inclusion bodies on wound healing. The IBs were used for coating of the cell culture surface or were directly added to the culture medium after wounding. Different modes of IB addition allowed the differentiation of texture effects promoting cell proliferation. It could be shown that the inclusion bodies of the epidermal lipoxygenase from the axolotl act as a reservoir of the functional enzyme and display a strong positive effect on wound closure in both experimental setups. Nevertheless, the exact mechanism of this effect is still unknown. For further study of the AmbLOXe enzyme it would be advantageous to use inclusion bodies, as well as soluble forms of the lipoxygenase, for the systematic screening of potential fatty acid substrates. Possible strategies for the soluble production of AmbLOXe include solubilization and refolding from bacterial inclusion bodies, or recombinant production from mammalian cell culture. In addition, ex vivo tissue models are the next logical step in investigating the efficacy of AmbLOXe in wound healing for human clinical applications.
